# Diagnosis of Early Pregnancy

**Published:** 1873-11

**Authors:** 


Diagnosis of Early Pregnancy.—Adolph Rasch, M.D., draws attention to an important symptom of pregnancy of the first three months, of which, until now, no notice has been taken by French, English and German authors. No opinion should be expressed in any case unless the uterus had been made out beyond doubt by the bimanual examination. The vaginal examinator should always be made by *two* fingers, unless circumstances forbade it, as by so doing results much more accurate could be obtained. An enlargement found, the distinction had to be made between enlargement by hypertrophy or by tumors, and enlargement by pregnancy. To solve this difficulty, the author has continued his investigations in a very large number of cases, of which he kept notes for nearly ten years, and enlarged experience has fully borne out what had helped him in making a few times a right diagnosis when better men had failed. This important symptom was fluctuation. That it must be felt very early, seemed to him *a priori* certain. For why should half an ounce or more of liquor amnii, enclosed under conditions very favorable for this purpose, not be felt fluctuating equally well as a few drops of pus in a panaritium? The notes of several hundred cases satisfactorily answer this question. Fluctuation could be felt in some cases as early as the seventh week of pregnancy; in most cases, after the second month. By adding to this the areolar signs of the *mammæ*, we should be able, in many cases, to make an almost certain diagnosis—considering the great variety of retained menses or other discharges, mistakes would be rare, even if other symptoms did not help us to make a distinction. The best way to feel it was to introduce two fingers into the vagina, while the other hand steadied the womb through the abdominal walls, and alternately to manipulate the uterus with the fingers. In some part of the uterus the fluctuation would be found, often in one corner of the fundus, sometimes lower down. The catheter should be introduced when accurate results were desired.

